# The Effect of Traditional Cupping on Pain and Mechanical Thresholds in Patients with Chronic Nonspecific Neck Pain: A Randomised Controlled Pilot Study

**DOI:** 10.1155/2012/429718

**Published:** 2011-12-07

**Authors:** Romy Lauche, Holger Cramer, Claudia Hohmann, Kyung-Eun Choi, Thomas Rampp, Felix Joyonto Saha, Frauke Musial, Jost Langhorst, Gustav Dobos

**Affiliations:** ^1^Chair of Complementary and Integrative Medicine, University of Duisburg-Essen, Knappschafts-Krankenhaus, Am Deimelsberg 34a, 45276 Essen, Germany; ^2^Department of Community Medicine, The National Research Centre in Complementary and Alternative Medicine (NAFKAM), Faculty of Health Science, University of Tromsø, 9037 Tromsø, Norway

## Abstract

*Introduction*. Cupping has been used since antiquity in the treatment of pain conditions. In this pilot study, we investigated the effect of traditional cupping therapy on chronic nonspecific neck pain (CNP) and mechanical sensory thresholds. *Methods*. Fifty CNP patients were randomly assigned to treatment (TG, *n* = 25) or waiting list control group (WL, *n* = 25). TG received a single cupping treatment. Pain at rest (PR), pain related to movement (PM), quality of life (SF-36), Neck Disability Index (NDI), mechanical detection (MDT), vibration detection (MDT), and pressure pain thresholds (PPT) were measured before and three days after a single cupping treatment. Patients also kept a pain and medication diary (PaDi, MeDi) during the study. *Results*. Baseline characteristics were similar in the two groups. After cupping TG reported significantly less pain (PR: −17.9 mm VAS, 95%CI −29.2 to −6.6; PM: −19.7, 95%CI −32.2 to −7.2; PaDi: −1.5 points on NRS, 95%CI −2.5 to −0.4; all *P* < 0.05) and higher quality of life than WL (SF-36, Physical Functioning: 7.5, 95%CI 1.4 to 13.5; Bodily Pain: 14.9, 95%CI 4.4 to 25.4; Physical Component Score: 5.0, 95%CI 1.4 to 8.5; all *P* < 0.05). No significant effect was found for NDI, MDT, or VDT, but TG showed significantly higher PPT at pain-areas than WL (in lg(kPa); pain-maximum: 0.088, 95%CI 0.029 to 0.148, pain-adjacent: 0.118, 95%CI 0.038 to 0.199; both *P* < 0.01). *Conclusion*. A single application of traditional cupping might be an effective treatment for improving pain, quality of life, and hyperalgesia in CNP.

## 1. Introduction

Neck pain, that is, pain between the occipital bone, the thoracic vertebra, and the extensions to the shoulder joint [1], is a major health-related socioeconomic problem and the lifetime prevalence is approximately 48.5% [2]. Neck pain can be caused by trauma, inflammatory diseases, or degeneration of the spine; however, most patients suffer from simple or non-specific neck pain, which is mainly caused by mechanical factors such as sprain and strains [3]. The aetiology of non-specific neck pain is not yet understood in detail, but different factors have been shown to contribute to the development and persistence of neck pain. They do not only include poor posture [3] and high physical load [4], but also poor psychological health [1, 5], stress [6], low socioeconomic status [7], and smoking [8, 9]. Usually non-specific neck pain resolves within three to six months; but 14% of the patients will suffer from recurrent or persistent pain [10]. If neck pain persists for more than 3 months, it is considered chronic neck pain [11].

Besides the pain and the related impairment in daily activities, chronic neck pain is also associated with functional changes. For example dysfunctional microcirculation of the trapezius muscle [12, 13] has been reported as well as motor control disturbances of the neck musculature [14]. Mechanical hyperalgesia, that is, increased response to painful mechanical stimulation has also been shown in chronic non-specific neck pain [15–17]; an effect which might be related to active trigger points, present in chronic non-specific neck pain patients but not in healthy controls [18]. However, it is still unknown if this process is restricted to the cervical area [16] or widespread [19]. Hyperalgesia in chronic non-specific neck pain also shows different patterns and seems to rely on different mechanisms than hyperalgesia in acute [19] and traumatic neck pain [15], respectively.

Although there is only limited evidence for these treatments, conventional treatment options include the prescription of nonsteroidal anti-inflammatory drugs [20], physical therapy [21, 22] or exercise [23, 24]. According to the literature [20, 25] and treatment guidelines [25] pharmacological therapy cannot be recommended, the same is true for manual therapy [26], or massages [21]. Dynamic and isometric exercises as part of physical therapy have also been proven to be only moderately effective in the long term [25]. Due to the limited treatment patients seek alternative treatment options, especially those patients with more intense pain [27, 28] and those who have not experienced improvements under conventional treatment [29].

Traditional cupping or wet cupping has been used in the treatment of pain and many other complaints for millennia [30]. A glass cup is utilized to create suction over a painful area after incisions are made to the skin. By doing so, it is hypothesised that “congested” blood is sucked out of the skin thereby increasing blood and lymphatic circulation and relieving painful muscle tension [30, 31].

Within the last years the interest in traditional cupping has emerged and there is growing evidence that cupping might be effective in various pain conditions [32–37]. Michalsen et al. [37] for example, found that a single traditional cupping treatment at the trapezius muscle was effective in relieving the symptoms of the carpal tunnel syndrome as well as associated neck pain. Lüdtke et al. [36] investigated the effect of traditional cupping in Brachialgia parasthetica nocturna, that is, numb, tingling, and painful sensations in fingers or hands during the night. A single treatment significantly reduced symptoms and the associated neck pain and no adverse events were observed. Farhadi et al. [34] found significantly reduced pain, functional disability, and pain medication in patients with low-back pain three months after traditional cupping compared to standard care. Cupping might further be effective in migraine and tension-type headache [32] and postherpetic pain [38]. However, despite growing evidence there is yet no RCT to investigate the effectiveness of traditional cupping in the treatment of chronic non-specific neck pain.

The aim of this pilot study was to test the efficacy of a single traditional cupping treatment in patients with chronic non-specific neck pain. Besides pain ratings we determined mechanical thresholds at pain-related and control areas to serve as more objective pain markers. We hypothesised that patients in the treatment group would report less pain at T2 compared to the waiting list control group.

## 2. Methods

### 2.1. Patients

The study protocol was approved by the institutional review board of the University Duisburg-Essen Medical Institutions (no. 09–3985). Between July 2009 and July 2010 50 patients aged 18 to 75 who suffered from neck pain for at least three months in a row with a minimum of 40 mm intensity on a 100 mm visual analogue scale (VAS) were included in the study. A specific inclusion criterion was based on the recommendations for traditional cupping [30, 39, 40]. Accordingly, patients show so-called plethora or overabundance. These terms refer to different signs and symptoms such as voluminous gelosis of the subskin, which indicates local blood congestion, swelling, and adhesions of the connective tissue in the neck region. A strong constitution, for example, high level of vitality, and high blood pressure, were further indicators for traditional cupping. Patients with blank myogelosis, that is, hyperirritable areas of skeletal muscle associated with small palpable nodules in taut bands of muscle fibres together with lowered microcirculation, were referred for dry cupping.

Patients were included only if neck pain was clearly identified to be of mechanical origin and specific causes for their neck pain had been excluded in the medical history either by an orthopaedist or a neurologist. Specific causes included traumatic neck pain (e.g., WAD), inflammatory or malignant disease, congenital malformation of the spine, radicular symptoms such as radiating pain, paresis, prickling, or tingling, invasive treatments within the last 4 weeks, surgery to the spine within the last year, and corticosteroid or opioid treatment. Further exclusion criteria were pregnancy, serious acute or chronic organic diseases such as diabetes or cancer, mental disorders, and haemorrhagic tendency or anticoagulation treatment. Nonsteroidal pain medication and physiotherapy were allowed if the treatment regimen was not altered for four weeks before and continued during the study. This ensured that statistical evaluation of the effects of cupping treatment was not influenced by alterations in medications or physiotherapy during the study phase.

All patients were recruited through advertisements in local newspapers and screened two times. First inclusion and exclusion criteria were checked in a standardised telephone interview, then the patients underwent a physical and neurological examination by the study physician at their first appointment.

### 2.2. Outcome Measures

#### 2.2.1. Pain

Pain at rest (PR) and maximal pain related to movement (PM, provoked pain by neck flexion, neck extension, lateral neck flexion, and neck rotation in either direction) [41] were recorded on a VAS graded from 0 (no pain at all) to 100 mm (worst pain imaginable). The minimal clinical important difference (MCID) for the VAS, a highly reliable instrument to measure pain intensity [42], is a reduction of 30%, which is the equivalent of a moderate pain reduction [43]. For PM the direction that elicited highest pain report was chosen for analysis. Baseline and postintervention pain scores were recorded at T1 and T2. Additionally patients kept a pain (PaDi) and medication diary (MeDi) from day 0 (7 days prior to T1) until T2, where they rated their pain three times daily on a 11-point numeric rating scale (NRS ranging from 0 = no pain to 10 = worst pain imaginable) and made notes of concurrent medication and treatments.

#### 2.2.2. Questionnaires

Self-rated disability due to neck pain was assessed with the Neck Disability Index (NDI) [44], a 10-item questionnaire representing everyday activities. The MCID for the NDI is 10% improvement for uncomplicated neck pain [45]. The health-related quality of life was quantified by the German version of the Medical Outcomes Study Short Form-36 (SF-36) [46, 47]. The SF-36 provides a detailed health profile on the basis of eight health dimensions as well as sum scores for physical and mental health. Two versions were used in the study, the standard version (4-week time frame) for baseline assessment at T1 and the acute recall version (1-week time frame) at T2. The latter version was used because it was considered more sensitive to recent changes in health status [48].

#### 2.2.3. General Health Outcome

Within the SF-36 the General Health outcome was recorded on a 5-point Likert scale that ranged from “My health is much better than before treatment” to “My health is much worse than before treatment.”

#### 2.2.4. Mechanical Sensory and Pain Thresholds

Sensory testing included determination of mechanical detection threshold (MDT), vibration detection threshold (VDT), and pressure pain threshold (PPT) and was conducted in the following areas: the site of maximal pain (pain-maximum), adjacent to the pain maximum (pain-adjacent), hand and foot on the right side. Pain-maximum and pain-adjacent were determined for each patient individually. First, the patient was given a diagram of the body on which to mark the most painful spot in the neck region. This spot, defined as pain-maximum, was verified by physical examination. The second spot, defined as adjacent to the painful area (pain-adjacent) was defined outside the painful area, that is, patients did not report pain in that area. Again physical examination was used to confirm the location. Both spots were marked in the pain diagram for precise replication of the measurements at T2. Thresholds were also determined at control areas, that is, right hand and right foot, in order to estimate reliability of measurements. All sensory measures were determined and calculated according to the standardised protocol for the quantitative sensory testing (QST) by Rolke et al. [49, 50], and MDT and PPT were logarithmised to reach normal distribution [49].

Mechanical detection threshold was measured with a set of von Frey filaments (Aesthesiometer, SOMEDIC, Sweden) that exert forces between 0.26 and 1080 mN. The threshold was determined by the method of limits, whereby the stimulus intensity is decreased until the patient can no longer perceive the touch and is then increased until the patient first perceives the touch again. Five series of descending and ascending stimulus intensities were made at pain-maximum, pain-adjacent, on the dorsa of the right hand and the right foot. The final threshold was the log-transformed geometric mean of these five series [49].

VDT was quantified by a Rydel Seiffer tuning fork (64 Hz, 8/8 scale). It was placed over a bony prominence, for example, on the spinal process, the styloid process of ulna and the lateral malleolus and left there until the subject could not feel the vibration anymore. The arithmetic mean of three series was taken the individual vibration detection threshold [49].

PPT was measured by a pressure algometer (Algometer, SOMEDIC, Sweden) at pain-maximum, pain-adjacent, the thenar eminence, and the instep. It exerts forces up to 2000 kPa when used with a probe area of 1 cm². The pressure pain threshold was measured in three ramps of increasing pressure intensities of ca. 50 kPa/s until the subject signalled the first sensation of pain in addition to the pressure sensation. The log-transformed arithmetic mean of these three series was taken the individual pressure pain threshold [49].

To evaluate the reliability of the sensory threshold measurements, the retest reliabilities were determined at the control areas in the control group participants (WL, *N* = 23). Pearson's correlation coefficients were *r* = 0.35 for MDT hand (*P* = 0.09), *r* = 0.66 for MDT foot (*P* = 0.001), *r* = 0.79 for PPT hand (*P* = 0.00001), *r* = 0.71 for PPT foot (*P* = 0.0001), *r* = 0.56 for VDT hand (*P* = 0.005), and *r* = 0.73 for VDT foot (*P* = 0.0001). The average correlation coefficients was *r* = 0.63 which indicates sufficient reliability.

#### 2.2.5. Safety

All participants were asked to report any adverse events during the study period. The questionnaires relating to T2 also included an open question about relevant experiences and adverse events.

#### 2.2.6. Expectation

After randomisation all patients had to self-rate their expectations towards cupping therapy on a VAS ranging from 0 = “not effective at all” to 100 mm = “most effective.”

### 2.3. Intervention: Traditional Cupping Technique

Based on data from previous studies on traditional cupping [36, 37] and clinical experience, a single cupping treatment was considered sufficient. Cupping was performed by the study physician, who was trained in cupping and regularly performed cupping in a clinical setting. Patients were asked to lay topless on the massage couch. The study physician used the patients pain diagram (see Section 2.2.4) and physical examination to identify the areas of pain and the voluminous geloses of the subskin, which most commonly were found at the descending parts of the trapezius muscle.

The cupping procedure involved the following steps: the skin was disinfected; superficial incisions were made with a disposable microlancet at the areas of pain and voluminous geloses; double-walled glass cups (2–6 glasses with diameters from 25 to 50 mm) were held inverted over an open flame to heat the air inside; the glass cup was placed on the incision. The air inside the cup cooled down and created a vacuum which sucked blood out through the incisions. The glasses were removed after 10 to 15 minutes, and the skin was disinfected and a plaster was applied. However, since bleeding generally stopped during treatment, this was only a precaution. Patients were asked not to take a bath or go swimming within the next 48 hours to prevent delays in wound healing. After some minutes of rest patients were free to leave.

### 2.4. Study Design

**Figure 1:**
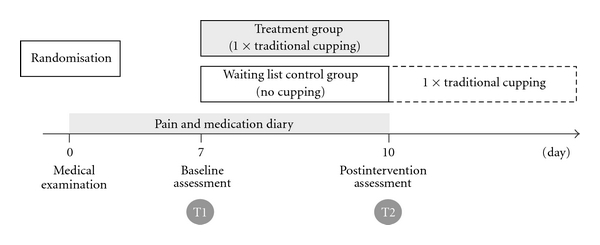
Study design.

After the telephone interview potential participants were invited to be assessed on whether they were eligible for the study. The study physician also informed them about the study details. Written informed consent was obtained and patients were then randomly assigned to either a treatment group or a waiting list control group by means of sequentially numbered, sealed opaque envelopes, prepared by the study coordinator, who was neither involved in treatment nor in measurement. Patients were handed out the pain and medication diary (PaDi, MeDi) and measurement and treatment appointments were scheduled.  Figure 1 illustrates the study design.

At baseline assessment (T1) study participants filled out the following questionnaires: pain at rest (PR), pain related to movement (PM), Neck Disability Index (NDI), and quality of life (SF-36). At last mechanical thresholds, that is, mechanical detection threshold (MDT), vibration detection threshold (VDT), and pressure pain threshold (PPT) were determined. At the end of T1 the treatment group received a single traditional cupping treatment whereas the waiting list control group received no treatment. Three days later participants returned for postintervention assessment (T2). They again filled out the questionnaires and underwent sensory testing. After they had completed the postintervention assessment, the wait-list control group was offered the cupping treatment.

### 2.5. Statistical Analyses

Treatment and waiting list control group were compared using chi-square analysis for discrete data and independent *t*-tests for continuous data on demographics, pain history, and baseline variables. For each outcome measure except the pain diary the results of the intervention were compared by analyses of covariance (ANCOVA) taking the post treatment measurement (T2) as a dependent and group as a between-subject factor. Respective baseline value of the outcome (T1) served as a covariate. This approach was chosen according to Vickers and Altman [51]. The intention-to-treat principle was used in this study, however, since all drop outs were lost before T1 missing data could not be replaced by taking the last observation forward.

The pain diary (PaDi) was analysed by means of a repeated measurement ANCOVA. Within the statistical model the group variable served as between-subject factor, the post intervention measures as dependent factors, and the average pain in the week before T1 as covariate. Medication and concurrent treatments (MeDi) were continuously recorded in the diary and converted into relative amount of days under medication or treatment.

The General Health outcome was analysed by means of the Mann-Whitney *U* test.

Because of the pilot character of the study the level of statistical significance was not adjusted. An alpha of 0.05 was chosen for all analyses.

## 3. Results

### 3.1. CONSORT Flowchart

After the first telephone screening, 122 patients were invited for further evaluation. 50 of them fulfilled the study criteria and agreed to participate in the study.

**Figure 2:**
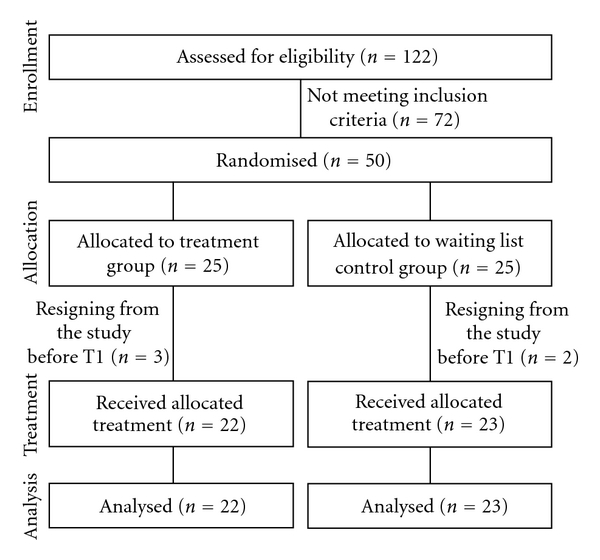
CONSORT flowchart of recruitment and study conditions.

Three patients in the treatment group and two in the waiting list control group resigned from participation before T1, no data could be collected from these patients. Final statistical analyses were conducted on 22 patients in the treatment group and on 23 patients in the waiting list control group.  Figure 2 shows a flowchart of patient recruitment.

### 3.2. Sample Characteristics

All baseline values were comparable between the two groups, see Table 1. Two-thirds of participants in the study were female, the average age was 54.8 (TG) and 57.2 (WL). Study patients suffered for a very long time from neck pain; on average they reported 12.0 (TG) and 10.4 (WL) years of pain. The average pain intensity was rated 44.9 (TG) and 42.6 (WL). Expectation was comparable between the groups; therefore it was not included in further analysis.

Pre- and postintervention scores and estimated differences are presented in Table 2 and described in detail below.

### 3.3. Pain

Analysis of pain at rest (PR) shows a significant group difference at T2. TG reported 17.9 mm less pain on the VAS than WL. There was also a significant group difference on maximal pain related to movement (PM). While both groups were comparable in their pain ratings at T1, TG reported 19.7 mm less movement-related pain than the WL at T2.

**Figure 3:**
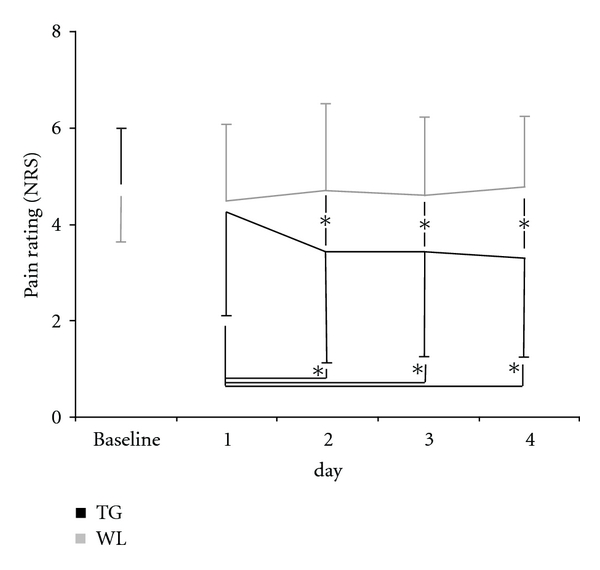
Pain ratings (pain diary, NRS, mean ± SD) decreased in TG at the day after cupping. *P < 0.05.

Pain diary (PaDi) shows a sudden decline in pain ratings in TG at day 2, that is, the day after cupping therapy whereas it remained relatively stable in WL (Figure 3). A repeated measures ANCOVA revealed a significant interaction time × group (*F* = 5.22, *Df* = 3/98, *ε* = 0.002, *P* = 0.005). Post hoc analyses confirmed a significant group difference at day 2 (Δ −1.5, 95%CI −2.5 to −0.4, *P* = 0.008) and single comparisons within TG also showed significant difference between baseline and day 2 (Δ −0.9, 95% CI −1.7 to −0.2, *P* = 0.014).

 The majority of the patients went without any concurrent treatment in the week before T1 (medication: 60.0%, physiotherapeutic treatment: 91.1%). Those who did, used medication in 27.8 ± 22.2 and physiotherapy in 39.3 ± 33.8 of the days. The use of medication and concurrent treatments during the study was not further analysed.

### 3.4. Questionnaires

No significant differences at T2 were found for the Neck Disability Index (NDI). The same was true for the Mental Component Score (SF-36) and the following subscales of the SF-36: Role Physical, Vitality, Social Function, Role Emotional, and General Health perceptions. On the other hand, significant differences occurred in the subscales Physical Functioning, Bodily Pain, and the Physical Component Score. At T2 TG reported significantly higher values on these scales indicating higher quality of life. Analysis of the General Health Outcome (SF-36) revealed a significant group difference with a significant higher rank for the TG (Mann Whitney *U* test; mean rank TG: 18.8; WL: 27.0; *U* = 160.5; *P* = 0.019) indicating more positive ratings than WL. In detail 11 patients of 22 in TG rated their health at least somewhat better than before, only two did so in WL. The majority in WL rated their health about the same as before (18 of 23). Three patients in each groups even reported worse health (see also “safety issues”).

### 3.5. Mechanical Sensory and Pain Thresholds

**Figure 4:**
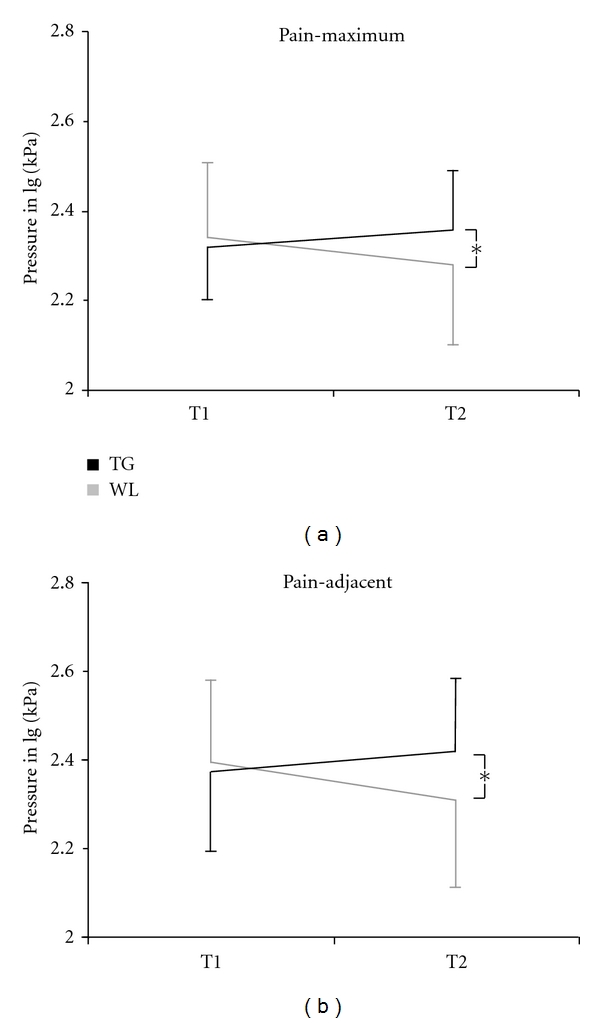
Course of pressure pain thresholds at pain-maximum and pain-adjacent (mean ± SD) *P < 0.05.

MDT, VDT, and PPT for each group are listed in Table 3. Statistical analyses revealed no group differences for MDT or VDT, but for PPT. Significant differences in pressure pain thresholds were found at the pain-maximum and the pain-adjacent, but not at the control areas.  Figure 4 displays the course of PPT at the pain-related areas.

### 3.6. Safety

Although most patients tolerated the treatment very well, adverse events were observed in some of the patients. One patient reported that the procedure itself was painful, other adverse events including slight reactions such as circulatory instability in the first minutes after treatment, tension headaches, a migraine attack, a reappearing tinnitus or wound healing itches. All of these adverse events were minor and transient.

However, two patients experienced more serious adverse events. As a result an ad hoc safety board was constituted to evaluate these adverse events and decide on further actions. The safety board was initiated by the principal investigator and consisted of the study physicians, the senior physicians of the Clinic for Complementary and Integrative Medicine, the head of the research group, an external statistician, and an external scientist, whose area of expertise is in safety issues and medical ethics. Two cases were presented and evaluated: one patient returned four days after treatment and reported worsened neck pain, a strong headache, and constant ear noises. The study physician examined the patient and diagnosed a cervical spine blockage. She was referred to an orthopedic for further diagnosis treatment. Later inquiries revealed that the symptoms had lasted for 2 to 3 weeks and improved subsequently. The neck pain, however, was neither better nor worse than before she participated in the study. Another patient complained of dizziness, nausea, and body misperception directly after treatment, so she had to lie down directly after treatment. Blood pressure and pulse measurement revealed normal circulatory function. The study physician diagnosed a transitory vagal reaction caused by the treatment and recommended her to rest until symptoms were resolved. After three hours lying and another hour sitting and walking the patient had mostly recovered. After examination the patient was sent home and a new appointment some days later was made. The patient then reported that the dizziness and nausea were fully resolved, but that the neck pain had worsened. The study physician offered her another treatment against the neck pain, which she refused. Later inquiries revealed that the pain had decreased within two weeks. The safety commission evaluated both incidents as adverse events, but not of a serious kind. Further actions as a consequence of occurrence of the adverse events involved obligatory follow-up check of patients in WL within two days after treatment. No adverse events were reported for WL after treatment.

## 4. Discussion

### 4.1. Principal Findings

To our knowledge, this is the first RCT where the effect of a single application of traditional cupping on chronic non-specific neck pain is investigated. Patients treated with cupping therapy showed significant improvements in their symptoms. Pain at rest (PR), maximal pain related to movement (PM), and bodily pain (SF-36) were reduced after a single cupping treatment. Pain diary (PaDi) showed a significant decline in pain ratings already on the day after cupping. According to the quality of life questionnaires (SF-36), the cupping treatment also significantly decreased Bodily Pain and improved Physical Functioning as well as the Physical Component Score.

Cupping also showed an effect on one of the nonsubjective parameters, the pressure-pain threshold (PPT), which is thought to reflect the functional status of (altered) pain perception. Pressure pain thresholds at pain-related areas increased or remained stable over time in the TG whereas patients of the WL control group became sensitised at those areas.

### 4.2. Interpretation

In this study various pain measures such as pain at rest (PR), pain related to movement (PM), and pain diary (PaDi) data differed significantly between the TG and the WL after cupping. Thus, a single traditional cupping treatment appears to be effective in treating chronic non-specific neck pain. Since changes in the VAS and the NDI were also strongly correlated (*r* = 0.49, *P* = 0.001, *N* = 45), pain relief appears to be associated with reduced impairment. However, there were no significant differences in NDI at T2 and the estimated difference was fewer than 10 points of improvement, which is the minimum clinical important change (MCIC) for the NDI [45]. This might have been due to the already low NDI scores at the beginning or due to the short followup. Interestingly, the pain diary ratings indicate that cupping has immediate effects. That is, the effects of traditional cupping are present already on the day after cupping treatment. This conforms to clinical observations, in which traditional cupping often shows dramatic and immediate effects on pain and other complaints.

Furthermore, Physical Functioning and the Physical Component Score (SF-36) changed significantly. These changes are impressive since the post intervention measurement was only four days after the treatment. However, the immediate pain relief and the changes on physical scales of the SF-36 suggest that traditional cupping might work on a very somatic level. Effects of cupping have also been found on pressure pain thresholds at pain-related areas. Although the differences in PPT were relatively small, they were found consistently, suggesting that cupping might exert its effects locally, probably at receptor level.

Different modes of actions might explain the effect of traditional cupping on chronic neck pain. They involve neural, haematological, immune, and psychological effects [34]. Stimulation of the skin causes several autonomous, hormonal, and immune reactions [52]; this also applies for injuries due to the incisions [53]. Blood vessels in the treated areas are dilated by release of vasodilators such as adenosine, noradrenaline, and histamine, which lead to increased blood circulation [54].

In the course of cupping treatment, blood and other interstitial fluids are drawn out from the skin by the vacuum. Traditional cupping is mainly used in patients with local blood congestion, swelling, and adhesions of the connective tissue in the neck region. It has been assumed that these congestions contain inflammatory extravasations [30, 35] and toxins. Cupping might therefore take the pressure off the tissue and relieve the neck area from these toxic congestions, which also increases circulation and lymphatic flow. Since circulation has been shown to be dysfunctional in chronic neck pain patients [12], cupping might restore normal circulation. Increased circulation in turn improves oxygen supply and cell metabolism [30] reducing the amount of inflammatory or toxic substances. This might also explain the significant effects of cupping on pressure pain thresholds at pain-related areas. Muscle spasm, congestion, and restricted blood flow can cause ischemic pain [55]. Accumulated inflammatory substances in skin and tissue might further induce hypersensitivity to noxious stimuli [16, 56], which is reflected by lowered pressure pain thresholds [57]. Since traditional cupping is supposed to evacuate toxins and inflammatory agents from the affected area and to restore normal circulation, this might explain the local effects on pressure pain thresholds.

The blood volume loss together with the local vasodilation might further increase parasympathetic activity by somatosympathetic reflexes, which corresponds well with the observed self-reported relaxation. Despite the invasiveness of traditional cupping the treatment group felt very relaxed after cupping treatment, on average they rated relaxation at 62.2 ± 20.1 mm on a 100 mm VAS from 0 = “not relaxed at all” to 100 mm = “very relaxed.” In the worst case the reflex might cause a vasovagal syncope, as observed in one patient.

### 4.3. Limitations of the Study

The interpretation of the results might be limited due to choice of the passive control group. We are aware of the fact that unspecific effects such as expectation, conditioning, or environmental effects may have contributed to the observed overall effect size [35]. However, to date there are no suitable sham devices [58], even though there is an urgent need for a suitable sham procedure. Sham cupping by means of adhesives to keep the glass in place have been tried, but in our experience even cupping naïve subjects are likely to discover the sham intervention, even more so in traditional cupping than in dry cupping. Another serious problem is the impracticability of experimental blinding the assessor due to superficial wounds and visible cupping marks. On the other hand, traditional cupping is applied commonly in clinical CAM settings and has been proven to be helpful in alleviating several pain conditions [34, 36, 37], and patients request this treatment. Therefore there is a need for clinical trials on the topic, evaluating the efficacy and safety of these procedures. In conclusion, instead of ignoring the fact that there is a patient request for this reasonably invasive procedure and that there is limited data on efficacy and safety available, we decided to run an RCT with the best possible methodological approach, even though we are aware of its limitations.

Expectation was high in the patients participating in this study, a fact which might reflect a selection bias. It is likely that only patients with high expectations took part in this study. However, baseline values were comparable between the groups.

A further limitation is the rather mild baseline pain intensity. Pain intensities reported by the patients in this study were at the lower end of the inclusion criteria scale. Some patients even fell below the required pain intensity of 40 on the VAS. This can be regarded as a possible source of bias, since patients probably exaggerated their complaints during screening to ensure inclusion into the study. The ceiling effect due to the low baseline pain intensity, likely limited the possible absolute reduction in pain intensity. Furthermore, due to the same problem, the likelihood of aggravation was also high due to the natural course of disease.

### 4.4. Strengths of the Study

Despite the limitations we could observe a strong pain reduction (VAS) of approximately 32.8 ± 51.1% in the treatment group, compared to 24.6 ± 88.7% in the waiting list control group. This pain reduction is comparable to studies on dry cupping [59] or massage [60] but in contrast du these methods the effect in traditional cupping occurs almost immediately after treatment. The overall pain reduction is within the range of clinical significance [43]. Moreover, treatment effects were also observed on pressure pain threshold, the concept of which is less transparent for the participant and therefore less open to presumption and hypothesising, which may make the results less biased than simple pain ratings.

## 5. Conclusion

A single application of traditional cupping might be effective in the treatment of chronic non-specific neck pain. Further studies are necessary to confirm these results and to evaluate the effectiveness of cupping compared to standard treatments. Studies investigating the effects of repeated traditional cupping interventions in different intervals and long-term observations are needed as well. Although measurements of sensory thresholds give possible hints on the physiology of pain processing, further investigations aiming at the mechanisms of action are necessary, too. However, the results of this first study and the patients' experiences with cupping therapy support the assumption that cupping might be a safe and effective treatment for chronic non-specific neck pain.

## Figures and Tables

**Table 1:** Baseline demographic and clinical characteristics of trial groups.

Sociodemographic and clinical characteristics	TG (*N* = 22) mean ± SD	WL (*N* = 23) mean ± SD	*P*
Age (years)	54.8 ± 9.6	57.2 ± 9.4	0.393
Sex (F/M)	18/7	16/9	0.544
BMI (kg/m²)	28.9 ± 5.6	27.1 ± 4.3	0.203
Pain at rest (PR)	44.9 ± 18.2	42.6 ± 17.8	0.810
Average neck pain at baseline (PaDi)	4.8 ± 1.1	4.6 ± 1.4	0.552
History of neck pain (years)	12.0 ± 10.3	10.4 ± 11.5	0.618
Expected effectiveness of cupping therapy (VAS from 0 = not effective at all to 100 = highly effective)	72.8 ± 18.9	68.3 ± 20.5	0.448

**Table 2:** Outcomes of subjective measures at T1 and T2.

	T1		T2		Estimated difference at T2	ANCOVA		
	TG (*n* = 22) (mean ± SD)	WL (*n* = 23) (mean ± SD)	TG (*n* = 22) (mean ± SD)	WL (*n* = 23) (mean ± SD)	diff TG versus WL* (95% CI)	*Df*	*F*	*P*
Pain at rest (PR)	44.9±18.2	42.6±17.8	28.5 ± 23.9	45.7 ± 16.4	−17.9 (−29.2 to −6.6)	44	10.2	**0.003**
Maximal pain related to movement (PM)	53.9±25.7	65.6±22.1	29.1 ± 20.9	53.8 ± 26.1	−19.7 (−32.2 to −7.2)	44	10.1	**0.003**
Neck Disability Index (NDI)	29.9 ± 11.8	31.1 ± 9.1	24.5 ± 13.5	29.0 ± 9.3	−3.6 (−8.7 to 1.6)	44	2.0	0.168
SF-36 Physical Functioning	74.5 ± 19.1	71.3 ± 20.7	80.0 ± 15.3	70.2 ± 19.2	7.5 (1.4 to 13.5)	44	6.2	**0.017**
SF-36 Role Physical	39.8 ± 37.5	39.1 ± 41.9	58.0 ± 41.8	51.1 ± 38.8	6.4 (−12.0 to 24.8)	44	0.5	0.483
SF-36 Bodily Pain	37.8 ± 9.3	39.7 ± 9.1	53.1 ± 22.9	39.3 ± 11.4	14.9 (4.4 to 25.4)	44	8.2	**0.007**
SF-36 General Health Perception	62.2 ± 14.2	64.0 ± 19.3	64.0 ± 14.8	61.3 ± 20.7	4.1 (−3.3 to 11.5)	44	1.3	0.268
SF-36 Vitality	59.5 ± 21.0	53.5 ± 19.6	61.4 ± 21.4	53.5 ± 23.8	2.1 (−5.1 to 9.2)	44	0.3	0.561
SF-36 Social Function	70.5 ± 25.7	69.6 ± 24.7	79.0 ± 26.6	73.9 ± 26.9	4.4 (−6.8 to 15.6)	44	0.6	0.434
SF-36 Role Emotional	81.8 ± 36.7	71.0 ± 39.3	81.8 ± 33.7	76.8 ± 39.5	−0.1 (−19.8 to 19.6)	44	0.0	0.991
SF-36 Mental Health	72.4 ± 15.9	68.2 ± 18.3	69.6 ± 21.4	68.5 ± 22.4	−3.4 (−10.7 to 4.0)	44	0.9	0.358
*SF-36 Physical Component Score *	37.8 ± 7.8	38.7 ± 8.6	43.3 ± 8.5	39.0 ± 7.4	5.0 (1.4 to 8.5)	44	7.8	**0.008**
*SF-36 Mental Component Score*	51.8 ± 10.8	48.7 ± 11.3	50.4 ± 11.7	49.8 ± 13.6	−2.1 (−7.1 to 3.0)	44	0.7	0.415

*Group differences and *P* values from an ANCOVA model with 2 groups, baseline values as covariate.

**Table 3:** Mechanical detection and pain thresholds at T1 and T2 (mean ± SD).

		T1		T2		Estimated difference at T2	ANCOVA		
		TG (*n* = 22) (mean ± SD)	WL (*n* = 23) (mean ± SD)	TG (*n* = 22) (mean ± SD)	WL (*n* = 23) (mean ± SD)	diff TG versus WL* (95% CI)	*Df*	*F*	*P*
MDT in log(mN)	Pain- maximum	0.425 ± 0.427	0.443 ± 0.418	0.446 ± 0.508	0.411 ± 0.433	0.047 (−0.185 to 0.278)	44		0.686
	Pain-sdjacent	0.290 ± 0.360	0.223 ± 0.374	0.382 ± 0.390	0.219 ± 0.477	0.124 (−0.094 to 0.341)	44		0.257

VDT in X/8	Pain-maximum	6.061 ± 1.542	5.986 ± 1.135	6.061 ± 1.398	5.956 ± 1.075	0.024 (−0.482 to 0.529)	44		0.447
	Pain-adjacent	5.288 ± 1.527	5.601 ± 1.162	5.515 ± 1.186	5.580 ± 1.401	0.199 (−0.366 to 0.764)	44		0.217

PPT in log(kPa)	Pain-maximum	2.349 ± 0.169	2.357 ± 0.192	2.381 ± 0.149	2.299 ± 0.192	0.088, (0.029 to 0.148)	44		**0.005**
	Pain-adjacent	2.396 ± 0.203	2.418 ± 0.200	2.423 ± 0.195	2.321 ± 0.204	0.118 (0.038 to 0.199)	44		**0.005**

Baseline values were comparable between the groups. *Differences were estimated by an ANCOVA model with 2 groups and the respective baseline values as covariate.
